# Italian multicenter study on infection hazards during dental practice: Control of environmental microbial contamination in public dental surgeries

**DOI:** 10.1186/1471-2458-8-187

**Published:** 2008-05-29

**Authors:** Paolo Castiglia, Giorgio Liguori, Maria Teresa Montagna, Christian Napoli, Cesira Pasquarella, Margherita Bergomi, Leila Fabiani, Silvano Monarca, Stefano Petti, SItI Working Group Hygiene in Dentistry

**Affiliations:** 1 Istituto di Igiene e Medicina Preventiva, Università degli Studi di Sassari, Via P. Manzella 4, 07100 Sassari, Italy; 2 Cattedra di Igiene ed Epidemiologia, Università degli Studi di Napoli "Parthenope", Via F. Acton, 38 – 80133 Napoli, Italy; 3 Dip. di Scienze Biomediche ed Oncologia Umana – Sez. Igiene, Università degli Studi di Bari, Piazza G. Cesare 11, 70124 Bari, Italy; 4 Dip di Sanità Pubblica, Università degli Studi di Parma, Via Volturno 39, 43100 Parma, Italy; 5 Dip. di Scienze di Sanità Pubblica, Università di Modena e Reggio Emilia, Via Campi 287 – 41100 Modena, Italy; 6 Dip. Di Medicina Interna e Sanità Pubblica – Cattedra di Igiene, Università degli Studi di L'Aquila, Via Vetoio 67010 Coppito di L'Aquila, L'Aquila, Italy; 7 Dip. di Specialità Medico Chirurgiche e Sanita' Pubblica, Università degli Studi di Perugia, via Del Giochetto, 06100 Perugia, Italy; 8 Dip. Scienze di Sanità Pubblica "G. Sanarelli", Università di Roma "La sapienza", Piazzale Aldo Moro 5, 00185 Roma, Italy

## Abstract

**Background:**

The present study assessed microbial contamination in Italian dental surgeries.

**Methods:**

An evaluation of water, air and surface microbial contamination in 102 dental units was carried out in eight Italian cities.

**Results:**

The findings showed water microbial contamination in all the dental surgeries; the proportion of water samples with microbial levels above those recommended decreased during working. With regard to *Legionella *spp., the proportion of positive samples was 33.3%. During work activity, the index of microbial air contamination (IMA) increased. The level of microbial accumulation on examined surfaces did not change over time.

**Conclusion:**

These findings confirm that some Italian dental surgeries show high biocontamination, as in other European Countries, which highlights the risk of occupational exposure and the need to apply effective measures to reduce microbial loads.

## Background

Infection hazards linked to dental practice are not a recent problem. Through this kind of health care practice, many infectious agents, both viruses (Hepatitis B virus, Hepatitis C virus, Human Immunodeficency virus, Herpes Simplex virus, Epstein Barr virus and *Cytomegalovirus*) and bacteria (*Streptococcus pneumoniae*, *Mycobacterium tuberculosis*, *Klebsiella pneumoniae*, *Escherichia coli*, *Legionella pneumophila *and *Pseudomonas aeruginosa*), may be transmitted. Some studies have shown that the environment (water, air and surfaces) can play an important role in this context: water stagnation, biofilm production and lack of disinfection in dental unit water systems (DUWS) promote the proliferation of microorganisms [1-4]. In addition, ablators, turbines and air-water syringes may nebulize the saliva and microorganisms contained in the patient's mouth, with the consequent contamination of the surrounding air and surfaces [5]. This suggests a potential risk of infection, especially from water, particularly in patients who are immunocompromised due to drug therapy, alcohol abuse, systemic diseases, and old age [6]. Some authors have declared that there is no evidence that dental unit water is harmful to patients, even though the lower the water contamination, the lower the risk to patients, and exposure to water that contains high numbers of bacteria violates basic principles of clinical infection control [7,8]. Moreover, there may be an occupational hazard because of much greater exposure of the staff [9-16]. Therefore, in order to minimize the hazards linked to dental practice, various cut-offs of total viable count (TVC) in dental unit water have been proposed. In 1995, the American Dental Association (ADA) stated that the TVC must be ≤ 200 CFU/mL (Colony Forming Unit), which was based on the quality assurance standard established for dialysate fluid [17]. The Centers for Diseases Control and Prevention (CDC) in their 2003 guidelines, reported that, for coolant water used in non-surgical dental procedures, the TVC must be ≤ 500 CFU/mL, as established by the US Environmental Protection Agency for drinking water and the ADA in 2007 [8,18].

A multicenter study that assessed the microbiology of DUWSs in general dental practice across seven European countries has shown that a substantial proportion have high levels of microbial contamination, which highlights the risk of occupational exposure and cross-infection during dental practice [19].

Italy did not participate in that study and no national data on this phenomenon have been reported; in particular, only a few papers on environmental microbial contamination in dental surgeries and cross-infection risk are present in literature [20,21]. Moreover, different methods were used in these studies.

The aim of this study was to evaluate the infection hazards linked to dental practice by checking the microbial contamination of water, air and surfaces in public dental surgeries in eight Italian cities.

## Methods

The study was carried out in 102 dental units (87 university and 15 public district facilities) at 64 dental clinics in eight Italian cities. Participation in the study was on a voluntary basis. All the participants collected specimens during the spring on the same Monday. Water specimens were collected from dental units (cup fillers and/or air-water syringes) at the start and end of morning practice (generally 08:00 and 13:00 h). Air and surfaces were sampled before the start (T_0_, empty room) and during clinical practice (T_1_, when the dentist, assistant and patient were present). In addition, on the same day, 91 water samples were collected from the washbasin taps at 08:00 h.

### Evaluation of water microbial contamination

All analyses were performed in conformity with Italian law [22], which complies with the European Council Directive 98/83/EC of 3 November 1998 [23]. The following parameters were determined:

- TVC after 7 days of incubation at 22°C and 5 days at 36°C using Yeast Extract Agar; in order to calculate the percentage exceeding safety levels, a threshold value of 20 CFU/mL at 36°C and 100 CFU/mL at 22°C was considered, in accordance with Italian law [22]; moreover, a threshold value of 500 CFU/mL was considered, as indicated by the CDC in 2003 [8];

- total coliforms presence after 24 h at 37°C and *E. coli *presence after 24 h at 44°C were determined by filtering twice 100 mL water through 0.45 μm membranes and using TTC Tergitol 7 agar medium; absence of both parameters in 100 mL was required [22].

In addition, the 87 university dental units were tested for *P. aeruginosa *and *Legionella *spp. Specimens were collected by mixing together equal volume of water from all water points of each dental unit (ablators, high- and low-speed handpieces, air-water syringes and cap fillers).

The presence of *P. aeruginosa* was detected by filtering 250 mL water through 0.45-μm membranes; these were then seeded on CN Pseudomonas agar and incubated at 37°C for 48 h. Absence of bacteria in 250 mL samples was required [22].

The presence of *Legionella spp.* was detected by filtering 1 L water through 0.2-μm isopore polycarbonate membranes; these were then resuspended in 10 mL of the same water sample and vortexed. Five mL was treated at 50°C for 30 min and seeded (0.1 mL) on cicloeximide, glicine, polymyxin, vancomycin (GVPC) agar medium. The remaining 5 mL was cold-seeded using the same technique. After incubation at 36°C for 8–10 days in a damp environment at 2.5% CO_2_, quantitative assessment was made, and expressed as CFU/L. Suspect colonies were subcultured on charcoal yeast extract (CYE) agar and buffered charcoal yeast extract (BCYE) agar and those ascribable to the *Legionella *genus were serologically identified [24].

All samples were collected in sterile bottles and immediately transported in a cool box (4–8°C) to the laboratory.

### Evaluation of microbial air contamination

Microbial air contamination was assessed by active sampling using the Surface Air System (SAS) to determine the number of CFU per m^3^, and by passive sampling using settle plates, 9 cm in diameter, exposed for 1 h, to determine the index of microbial air contamination (IMA) [25]. During the sampling, windows and doors were kept closed. The TVC was determined on Plate Count Agar.

The SAS was used to collect 180 L air in 1 min, near the dental unit, at 130–150 cm above the floor; after which plates were incubated for 48 h at 36°C, and the number of CFU was adjusted using the conversion table provided by the SAS producer and reported for 1 m^3 ^of air. The threshold value was assessed at 180 CFU/m^3^, as recommended for conventionally ventilated environments [26].

To determine the IMA, expressed as CFU/plate/h after 48 h at 36°C incubation, Petri dishes (diameter 9 cm) containing solid nutrient medium were left open to the air for 1 h at 1 m above floor level and at least 1 m from any relevant obstacle. A threshold of 25 was considered adequate [25,27].

Overall, 78 air samples were collected (46 using SAS and 32 using settle plates). As a rule, the air sampling was performed in the middle of each surgery, in a position near the head of the patient. When two or more samples were taken as results of surgery size, the mean value was used.

### Evaluation of surface microbial contamination

Microbiological analysis of the surfaces was performed using membrane filters (Sartorius SM 138 06 AC, ∅ 47 mm, 17.34 cm^2^, 0.45-μm pores) to determine microbial accumulation (MA) [28]. In order to calculate TVC, one sterile membrane was pressed with digital pressure for 30 s, using sterile gloves, on each surface to be controlled. The membrane was directly collected using sterile tweezers and placed on a sterile, dehydrated culture media previously moistened with 3 mL sterile distilled water. A total of 111 samples from the countertops and 110 from switches were collected. The results for MA were expressed as CFU/cm^2 ^after 48 h incubation at 36°C; a value of 1 CFU/cm^2 ^was considered acceptable [27].

### Statistical analysis

As regard dental units contamination, the statistical analysis of data was "machine oriented", i.e. no clustering analysis has been performed in case of more then one unit for dental surgery. Due to the skewed distribution of data, a logarithmic transformation was applied. Differences between transformed means of T_0 _and T_1 _counts were evaluated using Student's *t *test for paired comparisons. If a threshold was reached, prevalence was calculated as the proportion of values exceeding the cut-off. Independence between categorical variables was tested using Fisher's exact test. The McNemar test was applied for paired comparisons. The possible interference between the presence of *P. aeruginosa *and *Legionella *spp. was tested via Fisher's exact test. p < 0.05 was considered statistically significant.

## Results

TVCs at 22 and 36°C from water samples are shown in Table 1. T_0_-logarithmic means were higher than T_1 _at 22 and 36°C for cup fillers (1.6 vs 1.3, p = 0.16 and 1.7 vs 1.2, p = 0.02) and for air-water syringes (1.8 vs 1.4, p = 0.03 and 1.7 vs 1.2, p = 0.01). At the same time, the proportion of samples with higher levels than those recommended by Italian law was higher at T_0 _than at T_1 _at 22 and 36°C. In fact, these proportions decreased significantly at T_1 _for all parameters (McNemar test: p < 0.05), except for cup-fillers at 22°C, for which the reduction was not significant (McNemar test: p = 0.19). No samples exceeded the CDC 2003 threshold value of 500 CFU/mL (data not shown).

**Table 1:** Mean levels and standard errors (se) of TVC at 22° and 36°C (CFU/mL) from water samples, prevalence of samples with microbial levels above the threshold levels established by Italian law (21) (no samples exceeded the CDC 2003 threshold value).

	Cup fillers					
	
	22°C			36°C		
		
	T_0_	T_1_	Statist. anal.	T_0_	T_1_	Statist. anal.
Logarithmic mean	1.6 (se = 0.15)	1.3 (se = 0.14)	t = 1.38 (p = 0.16)	1.7 (se = 0.15)	1.2 (se = 0.13)	t = 2.24 (p = 0.02)
Geometric mean	42.7	21.9		47.9	17.4	
% positives (Italian law)	41.2 (42/102)	36.2 (37/102)	McN = 1.71 (p = 0.19)	60.8 (62/102)	47.0 (48/102)	McN = 10.32 (p = 0.001)

	Air-water syringes					
	
	22°C			36°C		
		
	T_0_	T_1_	Statist. anal.	T_0_	T_1_	Statist. anal.

Logarithmic mean	1.8 (se = 0.15)	1.4 (se = 0.14)	t = 2.11 (p = 0.03)	1.7 (se = 0.13)	1.2 (se = 0.13)	t = 2.55 (p = 0.01)
Geometric mean	64.6	23.4		49.0	16.6	
% positives (Italian law)	48.9 (47/96)	31.6 (30/95)	McN = 13.14 (p = 0.0003)	63.5 (61/96)	46.3 (44/95))	McN = 13.14 (p = 0.0002)

	Tap water					
		
	22°C	36°C				
			
	T_0_	T_0_				
				
Logarithmic mean	0.5 (se = 0.07)	0.7 (se = 0.10)				
Geometric mean	3.0	5.4				
% positives (Italian law)	4.4 (4/91)	29.3 (27/91)				

Statistical analysis of differences between T_0_ and T_1_ (t-test for paired samples for means and McNemar test for proportions)

The TVCs from tap water were lower than those from dental units at T_0 _(p < 0.001, data not shown). Total and fecal coliforms were not found.

Table 2 shows the relationship between *Legionella *spp. and *P. aeruginosa *isolated from 87 DUWSs. The prevalence of *P. aeruginosa *was 13.8%, while the prevalence of *Legionella *spp. was 33.3%. Only one sample (1.1%) was positive both for *P. aeruginosa *and *Legionella *spp., while 47 (54.0%) were negative for both microorganisms (one-tailed Fisher's exact test, p = 0.04) (Table 2).

**Table 2:** Prevalence of *Legionella *spp. and *Pseudomonas aeruginosa *in samples from dental unit waters

	*Pseudomonas aeruginosa*		
*Legionella *spp	Negative n. (%)	Positive n. (%)	Total n. (%)

Negative	47 (54.0)	11 (12.7)	58 (66.7)
Positive	28 (32.2)	1 (1.1)	29 (33.3)
Total	75 (86.2)	12 (13.8)	87 (100.0)

One-tailed Fisher's exact test p = 0.042

As regards air contamination, IMA was almost four times higher at T_1 _than at T_0 _(Table 3), with statistically significant differences between the two sampling times (p < 0.0001). Instead, the level of air contamination assessed by SAS did not change between T_0 _and T_1 _(from 104.7 CFU/m^3 ^mean value at T_0 _to 107.2 CFU/m^3 ^at T_1_, p = 0.39) (Table 4). The level of microbial accumulation on the countertops and switches of the dental units did not change over time (Table 5), with no significant differences between T_0 _and T_1_.

**Table 3:** Mean levels of air contamination assessed by IMA (TVC at 36°C, cfu/plate), prevalence of samples with microbial levels above the threshold level (25 CFU/plate)

	T_0_	T_1_	Statistical analysis
Logarithmic mean	0.8 (se = 0.11)	1.4 (se = 0.07)	t = 4.48 (p < 0.0001)
Geometric mean	6.5	23.4	
% positives	12.5 (4/32)	56.2 (18/32)	McN = 12.07 (p = 0.0005)

**Table 4:** Mean levels of air contamination assessed by SAS (TVC at 36°C, CFU/m^3^), prevalence of samples with microbial levels above the threshold level (180 CFU/m^3^)

	T_0_	T_1_	Statistical analysis
Logarithmic mean	2.0 (se = 0.05)	2.0 (se = 0.07)	t = 0.11 (p = 0.91)
Geometric mean	104.7	107.2	
% positives	39.1 (18/46)	34.8 (16/46)	McN = 0.75 (p = 0.39)

**Table 5:** Mean levels of microbial accumulation (TVC at 36°C, cfu/cm^2^) of operative surfaces (countertops and swiches), prevalence of samples with levels above the threshold level (1 cfu/cm^2^)

	Countertops			Switches		
		
	T_0_	T_1_	Stat. anal.	T_0_	T_1_	Stat. anal.
Logarithmic mean	0.01 (se = 0.08)	0.12 (se = 0.08)	t = 1.10 (p = 0.27)	-0.15 (se = 0.09)	0.005 (se = 0.09)	t = 1.23 (p = 0.22)
Geometric mean	1.0	1.3		0.7	1.0	
% positives	51.4 (57/111)	54.1 (60/111)	McN = 0.31 (p = 0.58)	48.2 (53/110)	52.7 (57/110)	McN = 1.8 (p = 0.18)

## Discussion

Some dental surgery environments in the eight cities involved in this study showed high levels of biocontamination. Water from oral rinsing cups and air-water syringes was already contaminated before work activity started, but the total microbial count never exceeded 500 CFU/mL (CDC 2003 threshold). The percentage of positive samples significantly decreased during the day (p < 0.05), especially in air-water syringes. Some authors [2,20] have suggested that this may be due to the presence of biofilms in DUWSs, as well as night-time stagnation, and in most cases, to the absence of adequate disinfection systems. Moreover, this seems to confirm that when water and air emission from syringes stops, a short depression sucks in external polluting particles [29].

Ineffective maintenance and disinfection of dental units probably causes *P. aeruginosa *colonization, as demonstrated by the percentage of positive water samples (13.8%). Moreover, *P. aeruginosa *can hide the presence of *Legionella *spp. [29]. Thus, even if the total microbial count does not always represent a risk for patients and health care workers, the presence of an opportunistic pathogen like *P. aeruginosa *may be dangerous, especially when associated with others microorganisms with a predilection for water habitats (e.g., *Legionella *and *Aeromonas *spp.) [29].

The water contamination results obtained in this study provide a contribution to the wider European investigation [19], which did not include Italian data. TVC was similar to the lowest reported counts, while the percentage of samples positive for *Legionella *spp. and *P. aeruginosa *was higher.

As to air microbial contamination, a high percentage of samples exceeded the limits of 25 and 180 for IMA and CFU/m^3^, respectively, whereas their mean values were lower than the threshold. IMA values increased during work activity (p < 0.0001), as expected, while CFU/m^3 ^values remained almost unchanged. Since an increase in air biological contamination caused by microbial dispersion from people and production of microbial aerosols is inevitable in dental practices, these results seem contradictory. A mutual relationship between the results obtained by active and passive sampling has been demonstrated in some studies [30-36]. Our study showed the need to promote further studies in this field.

As to surfaces, high microbial accumulation was registered at the beginning of work activities and increased during the day. More than 50% of samples showed values above the threshold. This was probably due to inadequate disinfection at the end of work activities and to the absence of proper aspiration systems, which caused a night-time fall-out of airborne particles. On the other hand, the contamination level does not exclusively depend on the number of patients and health operators, but also on failure to use films, blotting papers or trays to protect work surfaces on which instruments are placed during treatment, and which represent an indicator of contamination by aerosols, while the push-button panel indicates contamination due to manipulation.

An exhaustive analysis of microorganisms in air and surfaces was not completed, because the aim of this study was to propose a monitoring method for obtaining quantitative data, such as indicators of environmental hygienic conditions, leaving aside for the moment, identification of specific pathogens and the study of particular situations such as epidemic events.

## Conclusion

The control of indoor biological contamination is particularly important, as various pathogens may be transmitted from the environment to patients. Dental care is still a risk factor for several infections, especially in immunocompromised subjects [6]. Some authors [37] have insisted on the importance of individual protection systems and of frequent disinfection procedures in order to reduce the infection risk associated with dental aerosols, although the efficacy of these products varies over time [37]. In any case, it seems vitally important to promote staff training programs on the correct use of control procedures in work environments [8].

## Competing interests

The authors declare that they have no competing interests.

## Authors' contributions

The authors contributed equally to this work, read and approved the final manuscript.

## Pre-publication history

The pre-publication history for this paper can be accessed here:


